# Gelatin-hydroxyapatite nano-fibers as promising scaffolds for guided tissue regeneration (GTR): Preparation, assessment of the physicochemical properties and the effect on mesenchymal stem cells

**DOI:** 10.34172/japid.2020.001

**Published:** 2020-04-08

**Authors:** Simin Sharifi, Mohammad Samiei, Elaheh Dalir Abdolahinia, Rovshan Khalilov, Shahriar Shahi, Solmaz Maleki Dizaj

**Affiliations:** ^1^Dental and Periodontal Research Center, Tabriz University of Medical Sciences, Tabriz, Iran; ^2^Stem Cell Research Center, Tabriz University of Medical Sciences, Tabriz, Iran; ^3^Faculty of Dentistry, Tabriz University of Medical Sciences, Tabriz, Iran; ^4^Research Center of Pharmaceutical Nanotechnology, Tabriz University of Medical Sciences, Tabriz, Iran; ^5^Department of Biophysics and Molecular Biology, Baku State University, Baku, Azerbaijan; ^6^Institute of Radiation Problems, National Academy of Sciences of Azerbaijan, Baku, Azerbaijan; ^7^International Research and Education Center of Nanobiotechnology and Functional Nanosystems, Drohobych Ukraine & Baku, Azerbaijan

**Keywords:** Gelatin, GTR, Hydroxyapatite, Nano-scaffolds, Stem cell

## Abstract

**Background:**

Periodontitis can lead to progressive destruction of periodontal tissues supporting the tooth. Developing biomaterials for tissue engineering has noticeably improved the existing treatment options. The present study investigated the gelatin-hydroxyapatite nano-fibers as promising scaffolds for guided tissue regeneration (GTR).

**Methods:**

The scaffolds were prepared through electrospinning technique, and then the physicochemical properties and the cytotoxic effects on dental-derived mesenchymal stem cells were assessed.

**Results:**

The nano-scaffolds were successfully prepared with a mono-dispersed nano-scale diameter (102±0.10 nm), negative surface charge (-20±0.17 mV), and uniform network-shaped morphology. The mesenchymal stem cells derived from the human dental pulp stem cells (hDPSC) with gelatin-hydroxyapatite nano-fibers showed that the prepared scaffolds had a significant proliferative effect. Besides, the applied method can be used to prepare fiber-based structures via other polymeric materials.

**Conclusion:**

The incorporation of different materials to decrease the degradation rate of the fibers can match the speed of tissue regeneration. In this case, the prepared nano-fibers can be applied as a membrane biomaterial.

## Introduction

Periodontitis is characterized by the progressive destruction of periodontal tissues supporting the teeth. If untreated, periodontitis will cause tooth loss and aesthetic and phonetic problems. The structure of teeth is vital for the health of the oral cavity and the periodontium. The loss of teeth leads to many problems for the patient. Efforts are underway to regenerate lost teeth and osseous structures.^1^ Besides, peri-implantitis, as an inflammatory procedure of supporting marginal bone loss, mainly endangers the maintenance of dental implants.^2,3^ The conventional approaches used for peri-implantitis treatment are surgical and nonsurgical methods, including open flap debridement (OFD) or scaling and root planing (SRP), respectively.^4^

Xenografts, allografts, and autogenous and synthetic grafts (alloplasts) are four primary graft materials utilized in the clinics. Autograft resources, as grafts that are achieved from the same individual, are supposed to be the gold standard because they provide the perfect graft. The limitations of autografts include the possibility of a low bone volume, unpredictable resorption, and the possibility of contamination by the microorganisms in the oral cavity.^5,6^ Allografts obtained from the same species can be osteoinductive materials and osteoconductive scaffolds.^7^ Xenografts obtained from different species are extensively used in the clinic.

Recently, the periodontal regeneration research area has made significant progress in bone replacement grafts, soft tissue grafts, guided bone/tissue regeneration (GTR/GBR), modification of root surface, growth factors delivery, and gene therapy. A chief objective of periodontal regeneration is the regeneration of lost supporting tissues, including the alveolar bone, periodontal ligament, and cementum around a formerly unhealthy tooth root.^8,9^

Several types of materials are used in regenerative medicine. A perfect graft material should be non-toxic, biocompatible, non-immunogenic, and safe with no risk of disease transmission.^10^

Similar to a healthy tooth, the regenerated periodontal fiber must be orientated vertically to the alveolar bone and cementum. The epithelial cells form the long junctional epithelium during the healing period of periodontal therapy with the highest migration speed.^11^ Recently, some mixtures of current regenerative methods have been assessed, including hard tissue grafts, GTR, and the use of tissue growth factors.

One of the most extensively applied calcium phosphate-based graft materials is hydroxyapatite (HA) applied in both the clinical and research fields. They chemically bind to the bone when implanted due to their structure and composition similar to the minerals of natural bone.^12^ De novo formation of bone was detected mainly on the surface of HA without the interposition of fibrous tissue after the subcutaneous implantation of bone marrow stromal stem cells.^13^ The osteoblasts initiate the formation of partially mineralized osteoid, which is found on the surface of HA. It develops into a completely mineralized bone, causing firm bonding to the surface of HA. The effect of the synthetic hydroxyapatite was tested by Ogilvie et al^14^ in two adult patients with chronic periodontitis and tooth mobility. The results of the six-month implantation showed that the small crystals of biologic apatite appeared at the center of the synthetic hydroxyapatite aggregations. They were similar to those found in the adjacent alveolar bone and presented similar diffraction patterns. The radiological and clinical parameters, such as clinical attachment level (CAL), probing depth (PD), intrabony defect depth (IDD), and defect fill percentage, are commonly utilized to estimate periodontal regeneration. A nine-month study showed more regenerative effects with HA compared with an open flap debridement group.^15^

Owing to poor mechanical strength of inorganic materials, the organic constituent materials are used to control the seeding process of the inorganic materials.^16^ It has been reported that the biological response of inorganic nanomaterials can be superior to the combination of organic materials, such as collagen, gelatin, chitosan, etc.^17^ Collagen and HA have been used to prepare artificial scaffolds of bone substitutes. However, the significant limitations of collagen include the cost and unidentified commercial accessibility. A hydrolyzed derivative of collagen, gelatin, as a biodegradable polymer, can be utilized as an inexpensive and efficient alternative material. Gelatin is an appropriate material for the matrix of scaffold due to its similar sequence of amino acids. Furthermore, it eliminates the pathogen transmission risk and immunological problems of collagen.^18,19^

In this study, we prepared gelatin-hydroxyapatite nano-fibers as promising scaffolds for guided tissue regeneration (GTR). We also evaluated the physicochemical properties and the effect of the prepared scaffold on mesenchymal stem cells derived from the dental pulp.

## Methods

All the materials used (hydroxylapatite, gelatin, chloroform, dimethylformamide) were analytical grade and were procured from Sigma-Aldrich, Germany. The DPSCs were purchased from Shahid Beheshti University. Low-passage cells were used for the tests.

### Nano-fibrous scaffold preparation method

Gelatin was moderately dissolved (5% [w/v]) in chloroform/acetone mixture (80/20) using a magnetic stirrer. Then, HA was added (5.0% of gelatin [w/w]) into the prepared solution. The mixture was stirred at 45ºC for 30 minutes. The obtained polymer solution was then electrospun using an electrospinning machine (ANSTCo RN/I, Iran). The prepared polymer solution was located in a capillary tube (a 5-mL syringe) in a vertical form. A four-sided fixed collector was applied to collect the nano-fibers over the electrospinning jets. The electrospinning machine was set as follows: 20 kV voltage, a distance of 10 cm between a collector and a capillary tube, and a flow rate of 1.5 mL/h.

Scaffold Characterization

### The mean nano-fiber diameter

The mean nano-fiber diameter of the prepared scaffolds was calculated by the dynamic light scattering (DLS) process (Malvern, United Kingdom) at 25°C. The freshly prepared suspension of nano-fibers was diluted with distilled water and injected into the capillary cell of DLS.

### The morphology of the nano-fibers

The morphology of the gelatin-hydroxyapatite nano-scaffolds was tested by a scanning electron microscope (SEM, TESCAN, Warrendale, PA). The dry powder of the nano-fibers was fixed with adhesive tape and then sputter-coated with gold.

### Zeta potential measurements

Zeta potential measurements were performed by a zeta-sizer (Malvern, UK) at 25°C. The freshly formed suspension of nano-fibers was diluted with distilled water and inserted into the capillary cell of the machine.

### In vitro cytotoxic eﬀects of nano-scaffolds

Cell viability and cytotoxicity of the nano-scaffolds was tested by the MTT assay (3-(4, 5-dimethylthiazol-2-yl)-2,5-diphenyl tetrazolium bromide). In brief, hDPSC was treated on nano-scaffolds into 96-well plates at a density of 5000 cells/well. The plates were incubated at 37°C for seven days. Then, the MTT was added to each well to reach a ﬁnal concentration of 0.5 mg/mL. After four hours, the medium containing MTT was removed, and the purple formazan product was dissolved in 200 μL/well of dimethylsulfoxide (DMSO) and assessed at 570 nm. The cell viability associated with the control wells (cell culture medium without nano-scaffolds) was calculated by [A]test/[A] control ×100, with [A] as the concentration of viable cells.^21^

### Statistical analysis

SPSS 19 was used for statistical analysis. Statistical significance was set at P<0.05.

## Results and Discussion

**Figure 1:**
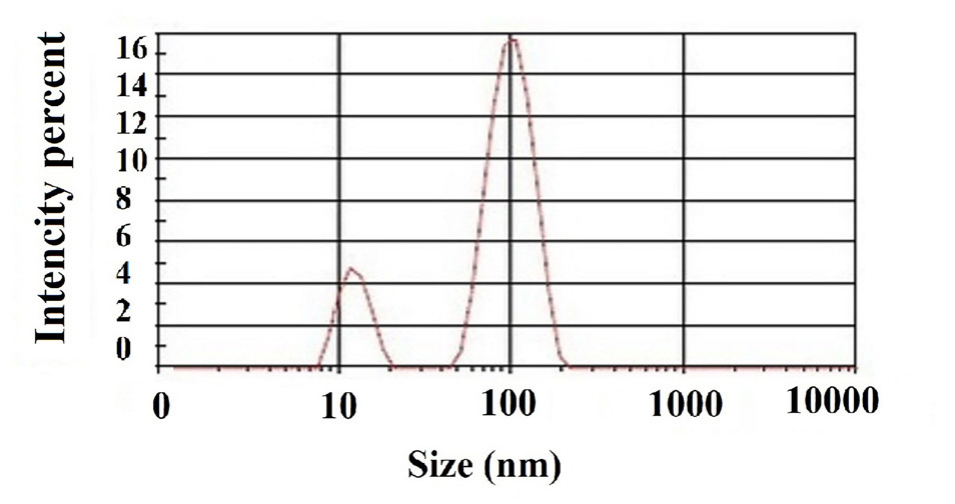
The mean fiber diameter of the prepared nano-scaffolds.

**Figure 2:**
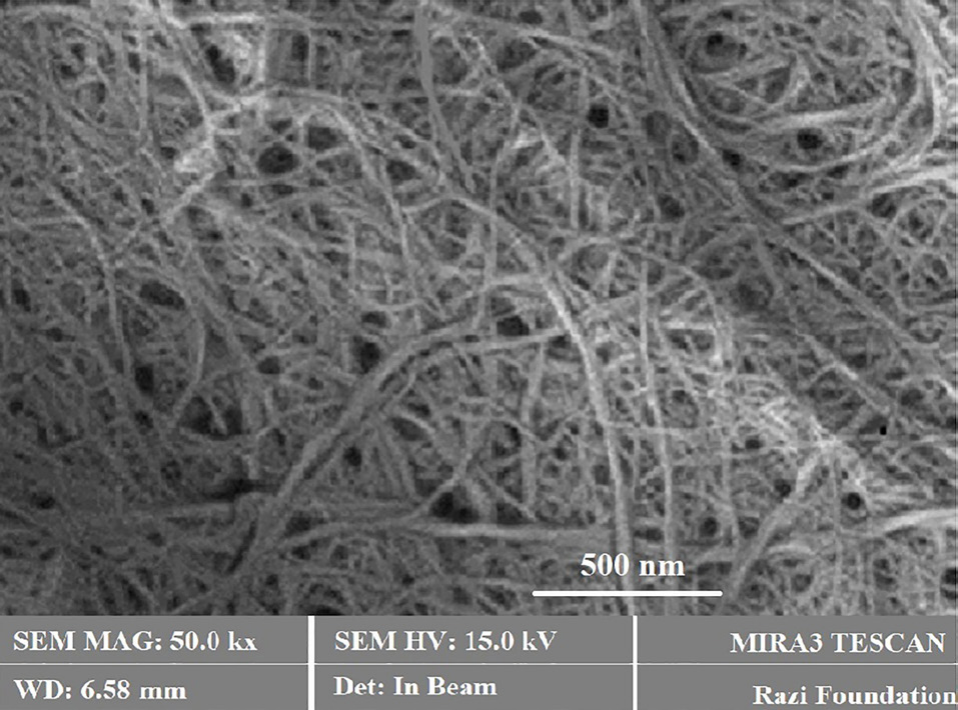
SEM image of the prepared nano-scaffolds.

The size and the shape of nanomaterials should be controlled, which is not a simple process. The growth of particles from atoms to nanometer or micrometric sizes can be controlled to produce mono-dispersed sizes. The physical properties change significantly with a change in the nano-scale diameters. For example, melting points drop dramatically with smaller nanoparticles. The mean particle size of the nanoparticles and the mean diameter of nano-fibers are key parameters that can affect different properties in biomedical applications.^20,21^ The mean diameter of the prepared fibrous nanomaterials was obtained as 102±0.10 nm with the polydispersity index (PDI) value of 0.23, which displays the relatively mono-dispersed nano-fibers (Figure 1). Other researchers have reported similar results for the nano-fibrous scaffolds.^22^ The structures of nano-fibers might also offer many fascinating topographies, such as outstanding mechanical possessions and highly specific surface areas, proposing them as attractive nanomaterials for many applications. Electrospinning, as a rapidly developing method, shows the capability to form diverse morphologies due to its flexibility, functionality, and simplicity. The optimum conditions in our work were: 20 kV voltage, a distance of 10 cm between the collector and capillary tube, with a 1.5 mL/h flow rate. We prepared non-cross-linked, biocomposite nano-fibrous scaffolds. The prepared fibers present a bridge-shaped morphology (Figure 2). It can also be observed from the SEM image that the nano-fibers were randomly electrospun on the collector with high similarity to the construction of the extracellular matrix (ECM). Indeed, natural ECM contains various protein fibers with size disparity from tens to hundreds of nanometers.^23^ The nanostructure of ECM support cells and can function as a helpful matrix to guide cell behavior.^24-26^ The nano-fibrous scaffolds have been shown to support the attachment, growth, and ECM remodeling of stem cells.^27^ In a study by Anjum et al,^28^ nano-fibrous biocomposites of poly(ε-caprolactone) and gelatin significantly enhanced the viability and proliferation of adult human skin-derived precursor cells (hSKPs). Their results also showed enhancement for the production of ECM in vitro and within skin wounds in vivo.^28^ Designing novel scaffolds with such architecture is one of the main challenges in the field of tissue engineering.^29,30^ Other reports have shown comparable results for the nano-fibrous scaffolds.^22,31^ Besides, the nano-fibers showed a network structure without the presence of beads. The diameters yielded by the DLS for the nano-fibers were different from the sizes found by the SEM images, which might be attributed to viscosity magnitudes in the Stokes–Einstein equation for the DLS method.^32^

**Figure 3:**
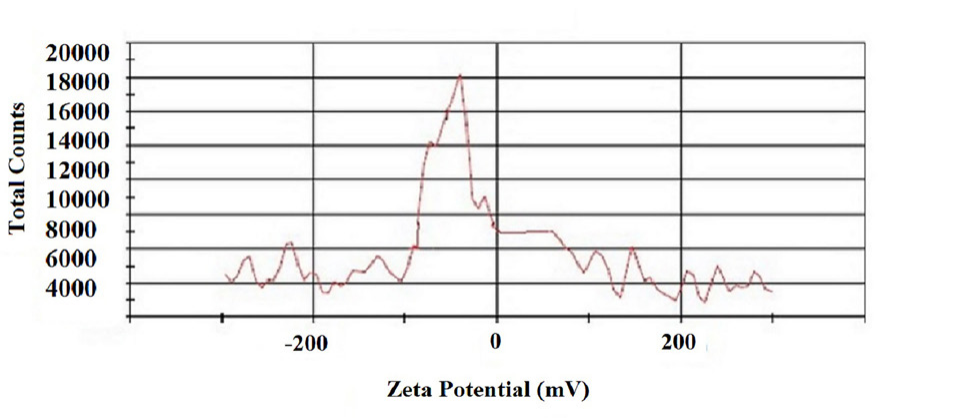
The average zeta potential of the prepared nano-scaffolds.

The mean zeta potential of the nano-fibers was -20±0.17 mV (Figure 3), representing fiber stability as the repulsive charge between the fibers to avoid agglomeration.^33,34^ It can also describe that the negative zeta potential has a significant and promising effect on the attachment and proliferation of the bone cells. Previous studies have shown that a surface with a negative charge is more accessible for the attachment and proliferation of osteoblasts than neutral or positive surface charges.^35,36^ These results establish that the processing parameters were effectively improved to prepare fine nano-fibers based on previous reports.

**Figure 4:**
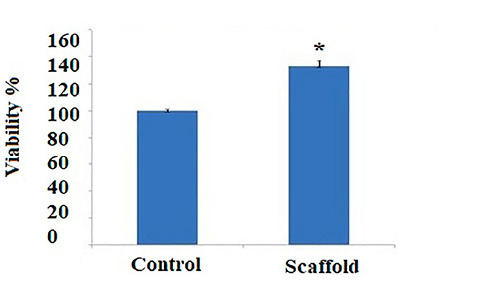
Cytotoxicity and cell viability of the nano-scaffolds by the MTT assay, Gelatin-hydroxyapatite nano-fibers displayed proliferative effect significantly (P<0.05).

Cytotoxicity examinations are critical before in vivo studies of biologically active compounds. In this study, cell viability and cytotoxicity of the gelatin-hydroxyapatite nano-fibers was studied by the MTT (Figure 4). Gelatin-hydroxyapatite nano-fibers showed a proliferative effect and no cytotoxicity. These results suggest the favorable cytocompatibility of the gelatin-hydroxyapatite nano-fibers.

Mobini et al^37^ prepared gelatin–hydroxyapatite nanocomposites via glutaraldehyde (GTA) as a cross-linking agent for the polymer. The MTT results suggest that the use of GTA in excess of 0.25% can be toxic to the cells. In the present study, non-cross-linked biocomposite nano-fiber membranes were prepared. Thus, it did not result in any toxicity related to the cross-linking agents. In another study, Nabipour et al^38^ tested the biocompatibility of a scaffold consisting of HA, PLA, and gelatin. MTT assay outcomes established the biocompatibility of the samples but did not have any proliferative effect.

Swain et al^39^ prepared hydroxyapatite (HA)–gelatin–polyvinyl alcohol (PVA) nano-scaffolds via three different spherical, rod, and fibrous HA nanoparticles. The in vitro cytotoxicity test showed cytocompatibility and cell viability at low extract concentrations up to 25%.

## Conclusion

In this study, the prepared scaffolds not only did have cytotoxic but also had a significant proliferative effect. Therefore, it can be a promising scaffold for periodontal regeneration. However, more in vivo studies are required. It is believed that by combining different materials to reduce the rate of fiber degradation, it is likely to complete the rate of tissue regeneration. Then, the prepared nano-fibers can be applied as a membrane biomaterial.

## Competing Interests

The authors declare no conflict(s) of interest related to the publication of this work.

## Authors’ Contributions

The study was designed by SMD. Data collection was carried out by SSH. SSH, EDA and SMD have made contribution to the research steps or the drafting and revision of the manuscript. MS, RK and SHS had contribution to the drafting and scientifically revision of the manuscript. SMD is the corresponding author of the manuscript. All authors read and approved the final manuscript.

## Ethics Approval

The ethics protocol was approved by the Ethics Committee in Medical Research, Tabriz University of Medical Sciences under the code IR.TBZMED.VCR.REC.1397.241.
